# The role of abdominal compression in the reduction of respiratory motion for cardiac radioablation

**DOI:** 10.1002/acm2.70561

**Published:** 2026-03-29

**Authors:** Jakob Marshall, Alanah Bergman, Tania Karan, Marc W. Deyell, Devin Schellenberg, Steven Thomas

**Affiliations:** ^1^ Department of Physics University of British Columbia Vancouver British Columbia Canada; ^2^ Medical Physics, BC Cancer Vancouver British Columbia Canada; ^3^ Department of Surgery Division of Radiation Oncology and Experimental Radiotherapeutics University of British Columbia Vancouver British Columbia Canada; ^4^ Centre for Cardiovascular Innovation and Division of Cardiology University of British Columbia Vancouver British Columbia Canada; ^5^ Radiation Oncology, BC Cancer Surrey British Columbia Canada

**Keywords:** abdominal compression, respiratory motion, stereotactic arrhythmia radioablation

## Abstract

**Background:**

Research on cardiac radioablation (CR) has highlighted the importance of accounting for respiratory‐induced motion during treatment, promoting investigation into methods to reduce its impact.

**Purpose:**

To investigate the efficacy of abdominal compression (AC) to reduce respiratory‐induced motion for CR patients by quantifying their respiratory‐induced motion under AC and free breathing (FB) conditions.

**Methods and materials:**

Nine patients were imaged with 5 Hz bi‐planar fluoroscopy on the Vero4DRT linear accelerator for 15–20 seconds under both AC and FB conditions in preparation for CR. As the soft tissue target is not visible on bi‐planar X‐ray images, the implantable cardioverter defibrillator (ICD) lead tip was used as a motion surrogate. The position of the ICD lead tip was tracked and triangulated in each bi‐planar image frame, with the respiratory component of motion extracted using a lowpass filter. Properties of respiratory‐induced motion were quantified including the average (across patient breaths) peak‐to‐peak motion extent, the mean total 3D magnitude of displacement, and the standard deviation of motion for each patient.

**Results:**

The average (95% CI) extent of RV lead respiratory‐induced motion was [3.0 (1.1, 5.0), 2.8 (1.6, 4.1), 7.0 (4.2, 9.7)] mm under FB and [2.1 (1.4, 2.8), 2.7 (1.7, 3.8), 4.7 (3.5, 5.9)] mm under AC in the [right–left, anterior–posterior, inferior–superior] directions. Eight out of nine patients had a reduction in the mean respiratory‐induced total 3D displacement under AC, with an average (± STD) across patients of (3.2 ± 1.7) mm under FB and (2.2 ± 0.8) mm under AC. Averaged across patients, the standard deviation (95% CI) of respiratory‐induced motion was [1.3 (0.5, 2.0), 1.2 (0.7, 1.6), 3.1 (1.7, 4.5)] mm under FB and [0.9 (0.6, 1.3), 1.2 (0.6, 1.8), 2.0 (1.3, 2.7)] mm under AC.

**Conclusions:**

For eight out of nine CR patients investigated, the respiratory‐induced RV lead motion was reduced when using AC compared to FB conditions.

## 1 INTRODUCTION

After failing conventional treatments, patients with ventricular tachycardia (VT), a rapid abnormal heart rhythm, often have limited options. The emergence of cardiac radioablation (CR), an experimental treatment for VT, may offer another option. CR, also termed stereotactic arrhythmia radioablation, or cardiac stereotactic body radiation therapy, delivers a single high dose (25 Gy) of radiation to the region in the heart responsible for VT. Promising results on the ability of CR to reduce VT burden has been presented.^1^, ^2^, ^3^, ^4^, ^5^, ^6^ However, the long‐term efficacy of the treatment remains to be fully established.^7^, ^8^, ^9^, ^10^, ^11^ Favorable patient outcomes are supported by enhancing treatment capabilities that ensure an appropriate delivery of radiation to the targeted region while minimizing radiation‐induced toxicities.

Maximizing the therapeutic potential of CR requires accurately delivering radiation to the moving heart. Motions due to the patients’ heart contraction and breathing must be understood and accounted for. Stevens et al. performed a literature review in 2023 concluding that detailed data on cardiorespiratory motion for CR is still limited.^12^ Several studies, both from the review paper and others published since have described the amplitude of cardiac motion for CR patients.^13^, ^14^, ^15^, ^16^, ^17^, ^18^, ^19^ These cardiac motions vary based on the region of interest in the left ventricle and the patient's heart function.^20^ The amplitude of respiratory motion, as measured at the heart has also been investigated, quantified by itself ^13^, ^14^, ^16^ or in conjunction with the cardiac motion.^21^ It has been shown that cardiorespiratory motion can induce substantial dose uncertainties, with the impact of respiratory motion greater than that of cardiac.^17^ This motivates better understanding respiratory motion in CR and methods to mitigate it.

A common approach used in radiation oncology to minimize patient breathing is abdominal compression (AC). AC uses a belt or plate to compress the abdomen, in an attempt to physically restrict the diaphragm motion, thus reducing respiratory motion. The impact of AC in CR is beginning to materialize, with two studies quantifying motions of the heart using respiratory 4DCT imaging.^22^, ^23^ Mannerberg et al. analyzed 18 lung cancer patients, imaged with and without AC, finding that AC has the potential to reduce respiratory‐induced motion for VT patients.^22^ Cecchi et al. found that AC was not a consistent motion management method for CR by comparing the heart's motion for 18 lung cancer patients under AC, 18 liver cancer patients under free breathing (FB) conditions, and 3 VT patients imaged under both AC and FB conditions.^23^ The disagreement between these works motivates further investigation into the efficacy of AC. Such investigation ideally includes a population of CR patients, imaged under both AC and FB to observe the individual effect within the target population. Further, to mitigate the effects of cardiac motion and variations in patient breathing it is desirable to analyze data of a sufficiently high temporal resolution to filter out cardiac motions, while acquiring data over multiple patient breaths.

Resolving respiratory motion of the heart over multiple patient breaths is challenged by the ability to image with a temporal resolution to decouple cardiorespiratory motion. While this is possible with magnetic resonance imaging, metal artifacts can arise from cardiac leads that VT patients have implanted from prior treatment interventions.^24^, ^25^ Further, planar X‐ray imaging is challenged in its ability to localize the radiolucent cardiac target. However, recent work has shown that the respiratory motion of implanted cardiac leads are correlated with one another and the diaphragm,^26^ making them a potential surrogate for the heart's respiratory motion. Further work is needed to establish the use of cardiac leads as a surrogate, and should only be used as a respiratory motion surrogate with caution. However, bi‐planar fluoroscopic imaging of these cardiac leads is advantageous as it provides temporal resolution not realizable with conventional volumetric imaging modalities (eg CT). This work uses bi‐planar fluoroscopy to assess the respiratory motion of cardiac leads under AC and FB conditions to investigate the effectiveness of AC to limit respiratory motion of the heart for CR.

## 2 METHODS

### 2.1 Patient data

Nine patients underwent a target motion assessment—a standard practice at our institution for targets prone to motion—in preparation for CR treatment as part of an institutional review board (IRB) approved study [H19‐03173]. Patients were positioned with their arms above their head on a carbon fiber overlay board with a custom mold cushion. An inflatable AC belt was surrounding the patient, placed just below the rib cage (Radiation Products Design Inc, USA). When in use, the belt was inflated to a pressure that was firm, but tolerable by the patient (∼20–40 mm Hg). Each patient was imaged twice, first under FB conditions followed by AC. Each image series acquired sagittal and coronal image projections using 5 Hz bi‐planar, kV fluoroscopy for 15–20 seconds (∼3–5 breaths) using the integrated imaging system on the Vero4DRT linear accelerator (Brainlab AG) with kV/mAs parameters chosen based on patient factors. The FB image set was acquired first, followed by an AC image set with the inflatable and adjustable compression belt.

**FIGURE 1:**
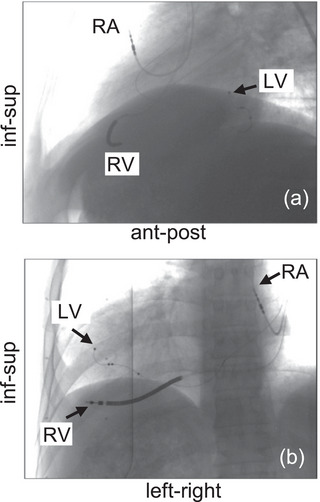
Bi‐planar views of the implanted cardiac leads monitored in this study.

As the soft tissue target is not visible on planar X‐ray images, the position of all implanted cardiac leads was tracked to assess respiratory motion using an in‐house MATLAB (MathWorks Inc) image processing application. All patients had an implantable cardioverter defibrillator (ICD) lead in the right ventricle (RV), termed the RV lead. The distal tip of this lead was semi‐automatically tracked on each orthogonal fluoroscopy image frame using normalized cross correlation image template matching. The lead's detected position, in each frame and orthogonal image, was verified and manually adjusted, if needed. The 3D position of the lead's tip was triangulated using the orthogonal projections for each simultaneous frame of image acquisition from the fluoroscopic imaging. This yields the 3D motion of the RV lead's tip throughout the image acquisition time. In addition to the RV lead, some patients had cardiac leads implanted in the (8 patients) right atrium (RA lead) and/or the surface of the (6 patients) left ventricle (LV lead). These leads, shown in Figure 1, were also tracked and triangulated to further understand the respiratory motion of different regions in the heart.

### 2.2 Respiratory motion extraction

The 3D motion of the cardiac leads comprises motion from the heart's beating (cardiac motion) and patient breathing (respiratory motion). As the purpose of AC is to restrict and hopefully reduce the motion from patient breathing, we extract the respiratory component of motion. This is enabled as the cardiac and respiratory motions occupy different ranges in the frequency domain. The more rapid, ∼1 Hz cardiac motion occurs at a higher frequency while the gradual respiratory motion, with respiratory periods of about 3–4 s, occurs at a lower frequency. Hence, the respiratory component of motion can be extracted using a low pass filter.^16^ The standard deviation of the combined cardiorespiratory lead motion and the extracted respiratory‐induced motion are calculated to compare the influence of these physiological processes. The respiratory motion is further analyzed to investigate the efficacy of AC to restrict respiratory motion.

To investigate the variability of patient breathing within the 15–20 seconds of image acquisition, the respiratory‐induced motion is separated into individual patient breaths. This is performed using the inferior‐superior (IS) motion of the diaphragm using the time‐point of end‐inhalation and end‐exhalation by manually defining the local extremum. Individual breathing cycles were then defined starting at end‐inhalation or end‐exhalation, depending on which occurred first during the image acquisition time. This breath definition was used for the motion of each cardiac lead, to define consistent breathing cycles.

### 2.3 Motion analysis

Various metrics were calculated to compare respiratory‐induced motion under AC and FB conditions for each of the patients’ implanted cardiac leads. First, the standard deviation of respiratory‐induced motion for each patient (across the entire 15–20 s of image acquisition) was calculated. Second, distributions of motion under AC and FB were compared using violin plots. Third, the peak‐to‐peak extent of respiratory‐induced motion was measured for each breath using the minimum and maximum position of the cardiac lead's respiratory‐induced motion within each breath. An average respiratory‐induced motion extent was calculated for each patient by averaging over patient breaths. The analysis was performed in the right–left (RL), anterior–posterior (AP), and IS directions. In addition, the magnitude of the vector displacement from the time‐averaged mean position was analyzed $\sqrt{RL^{2}+AP^{2}+IS^{2}}$. Herein, this magnitude of vector displacement is referred to as the total displacement.Statistical testing between AC and FB was not performed due to the limited sample size resultant from the experimental nature of CR treatment.

## 3 RESULTS

### 3.1 Respiratory motion extraction

**FIGURE 2:**
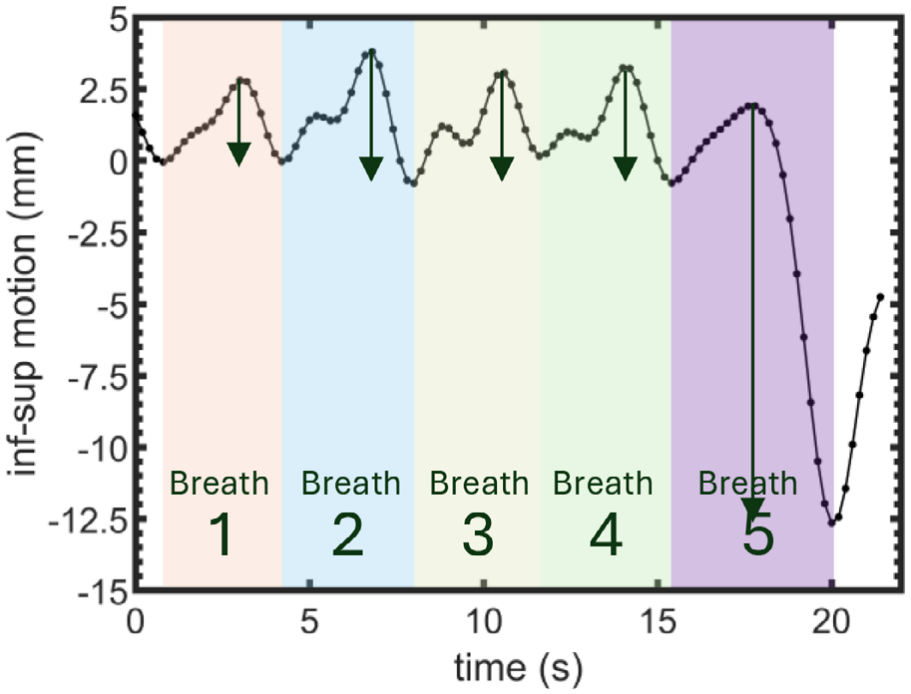
Definition of individual breaths and their (arrow) amplitude of respiratory motion. Data is shown for patient 4 under abdominal compression.

An example patient respiratory motion trace (patient 4 AC) is shown in Figure 2. Also illustrated is the definition of each patient breath and the measured peak‐to‐peak extent for each breath. This patient had a single, deep breath at the end of image acquisition that illustrates the impact of breathing variability on assessing motion management strategies.

**FIGURE 3:**
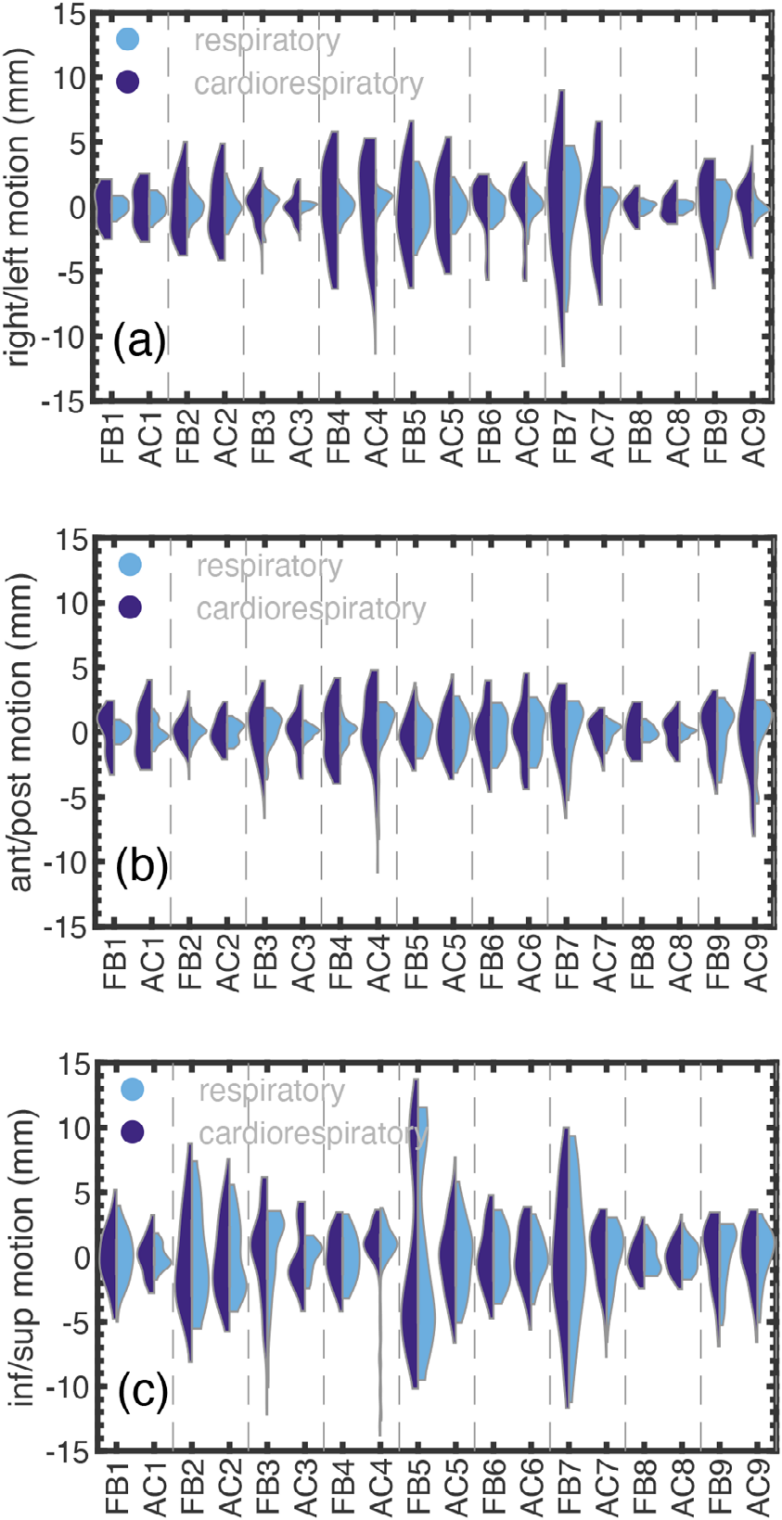
(Left half‐violin) cardiorespiratory motion and (right half‐violin) respiratory motion of the RV lead for each patient (1–9) under free‐breathing (FB) and abdominal compression (AC).

The distribution of each patient's motion for the RV lead is illustrated in the violin plots of Figure 3. The cardiorespiratory motion (left‐half‐violin) is shown alongside the extracted respiratory motion (right‐half‐violin), illustrating the contribution of this physiological process. The contributions of the cardiac and respiratory‐induced motions are quantified by the standard deviations of the RV lead motion. Under FB, the standard deviation (95% CI) of the RV lead cardiorespiratory motion was [2.4 (1.5, 3.3) RL, 1.8 (1.5, 2.2) AP, 3.4 (2.0, 4.7) IS] mm compared to [1.3 (0.5, 2.0) RL, 1.2 (0.7, 1.6) AP, 3.1 (1.7, 4.5) IS] mm for the respiratory component of motion. Similarly, under AC, the standard deviation (95% CI) of cardiorespiratory RV lead motion was [2.2 (1.4, 3.0) RL, 2.0 (1.3, 2.6) AP, 2.4 (1.8, 3.0) IS] mm and the standard deviation of respiratory‐induced RV lead motion was [0.9 (0.6, 1.3) RL, 1.2 (0.6, 1.8) AP, 2.0 (1.3, 2.7) IS] mm. The standard deviation for the respiratory‐induced motion of all cardiac leads, for each patient, is illustrated in Table 1.

**TABLE 1:** For each patient under abdominal compression (AC) and free breathing (FB), the number of breaths, average extent and standard deviation of the right ventricle (RV), left ventricle (LV), right atrium (RA) lead respiratory‐induced motion in the right‐left (RL), anterior‐posterior (AP), inferior‐superior (IS), and for the total (tot.) displacement trace $\sqrt{RL^{2}+AP^{2}+IS^{2}}$ is tabulated. The population average and standard deviation (STD) are shown in the last rows.

Pt.	No. Breaths	RV Extent (mm)				LV Extent (mm)				RA Extent (mm)				RV STD (mm)			LV STD (mm)			RA STD (mm)		
		RL	AP	IS	Tot.	RL	AP	IS	Tot.	RL	AP	IS	Tot.	RL	AP	IS	RL	AP	IS	RL	AP	IS
AC1	3	1.8	2.4	2.6	3.1									0.7	0.8	0.9						
FB1	3	1.6	1.5	5.4	5.5									0.5	0.5	1.9						
AC2	5	2.9	1.4	6.1	5.9	2.0	1.9	7.6	7.5	3.1	2.8	5.2	5.4	1.1	0.7	2.8	0.9	1.0	3.6	1.2	1.1	2.7
FB2	5	2.7	2.1	8.6	8.2	3.9	2.4	9.5	9.3	4.9	3.9	6.8	7.0	1.0	0.7	3.6	1.3	0.8	3.8	1.8	1.2	2.8
AC3	2	0.5	1.5	4.0	3.8	1.5	2.6	5.7	5.7	2.2	1.5	2.1	2.1	0.4	0.5	1.2	0.4	0.7	1.9	0.7	0.6	0.6
FB3	3	1.4	1.3	4.7	4.3	0.8	1.8	7.1	7.1	3.6	1.3	3.8	5.0	1.0	1.4	3.3	1.2	1.0	5.0	1.2	1.0	2.1
AC4	5	2.3	4.0	5.6	7.0	1.2	3.7	8.3	9.0	1.5	1.6	5.4	5.4	1.7	2.4	3.5	0.7	2.2	5.2	0.6	0.7	3.2
FB4	7	1.6	1.8	4.1	4.0	0.9	1.4	4.4	4.3	1.2	1.6	2.4	2.5	0.8	0.9	1.7	0.5	0.6	1.9	0.6	0.9	1.1
AC5	4	3.2	4.1	7.4	7.1					2.4	2.3	6.2	4.1	1.1	1.5	2.7				0.8	0.8	2.1
FB5	3	3.7	3.1	10.9	11.4					4.1	1.7	6.0	5.9	1.8	1.2	6.8				1.6	1.0	4.6
AC6	3	2.4	4.6	4.6	4.8					2.5	2.5	3.4	3.3	1.0	1.6	1.7				1.0	1.2	1.3
FB6	5	2.0	3.9	5.6	5.9					2.5	1.8	3.5	4.2	0.9	1.4	2.0				0.9	0.7	1.3
AC7	4	2.9	1.7	5.4	6.1	0.8	1.3	8.0	7.5	1.9	0.7	3.3	2.5	1.2	0.7	2.3	0.4	0.5	3.2	0.8	0.4	1.4
FB7	3	9.0	5.3	14.0	17.4	3.6	3.2	18.3	18.6	4.8	1.7	9.1	9.7	3.5	2.0	5.2	1.2	1.3	6.3	1.8	0.7	3.1
AC8	4	0.9	1.0	2.7	2.8	2.6	3.0	2.1	3.3	1.6	1.3	1.5	1.4	0.3	0.3	1.0	1.1	1.1	0.8	0.6	0.4	0.5
FB8	4	1.0	1.3	2.9	2.9	0.8	2.8	3.3	3.0	2.3	1.4	2.5	2.3	0.3	0.5	1.1	0.3	1.0	1.4	0.8	0.5	1.0
AC9	5	1.7	3.8	3.9	5.5	2.8	4.3	3.6	6.1	2.2	2.7	5.5	6.2	0.8	2.3	2.0	1.1	2.3	2.2	0.9	1.7	2.7
FB9	3	4.3	5.3	6.4	9.2	2.9	6.2	7.6	10.2	2.2	4.5	7.5	8.5	1.4	1.8	2.3	1.0	2.1	2.8	0.8	1.5	2.6
AC Av.	3.9	2.1	2.7	4.7	5.1	1.8	2.8	5.9	6.5	2.2	1.9	4.1	3.8	0.9	1.2	2.0	0.8	1.3	2.8	0.8	0.9	1.8
AC STD	1.1	0.9	1.4	1.6	1.6	0.8	1.1	2.6	2.0	0.5	0.7	1.7	1.8	0.4	0.8	0.9	0.3	0.8	1.5	0.2	0.4	1.0
FB Av.	4.0	3.0	2.8	7.0	7.6	2.1	3.0	8.4	8.7	3.2	2.2	5.2	5.6	1.3	1.2	3.1	0.9	1.1	3.5	1.2	0.9	2.3
FB STD	1.4	2.5	1.6	3.6	4.5	1.5	1.7	5.3	5.6	1.4	1.2	2.5	2.7	0.9	0.6	1.8	0.4	0.5	1.9	0.5	0.3	1.2

### 3.2 Motion analysis

**FIGURE 4:**
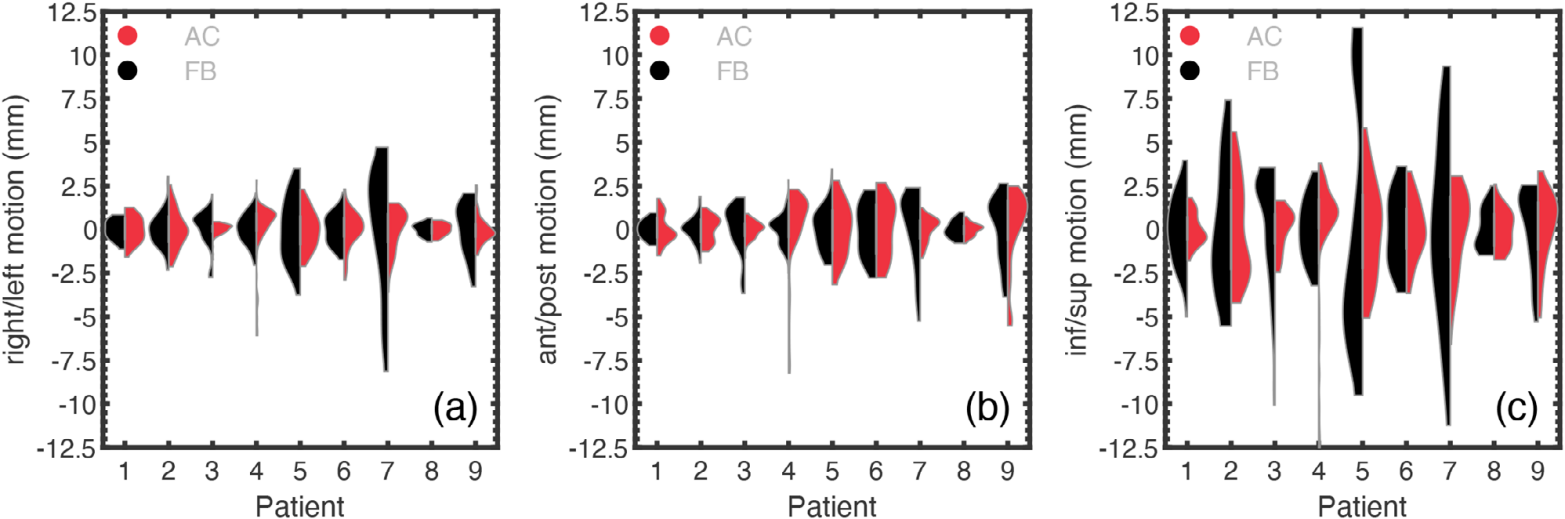
Respiratory motion of the RV lead for each patient under (black, left‐half‐violin) free‐breathing (FB) and (red, right‐half‐violin) abdominal compression (AC) conditions in the (a) right‐left, (b) anterior‐posterior, and (c) inferior‐superior directions.

The distributions of respiratory‐induced RV lead motion under AC and FB are compared in Figure 4. For each patient, the predominant direction of respiratory‐induced motion is in the IS direction. Also observed is the variability in the respiratory‐induced motion between patients, with each patient having different magnitudes and directions of motion. The ability of AC to reduce respiratory‐induced motion depends on the direction, as, for example, motion reductions in one direction can be partially offset by increased motion in an orthogonal direction. For example, patient 1 has reduced IS motion under AC but with increased motion in the RL and AP directions. Some patients, such as patient 7, have reduced motion in each direction.

**FIGURE 5:**
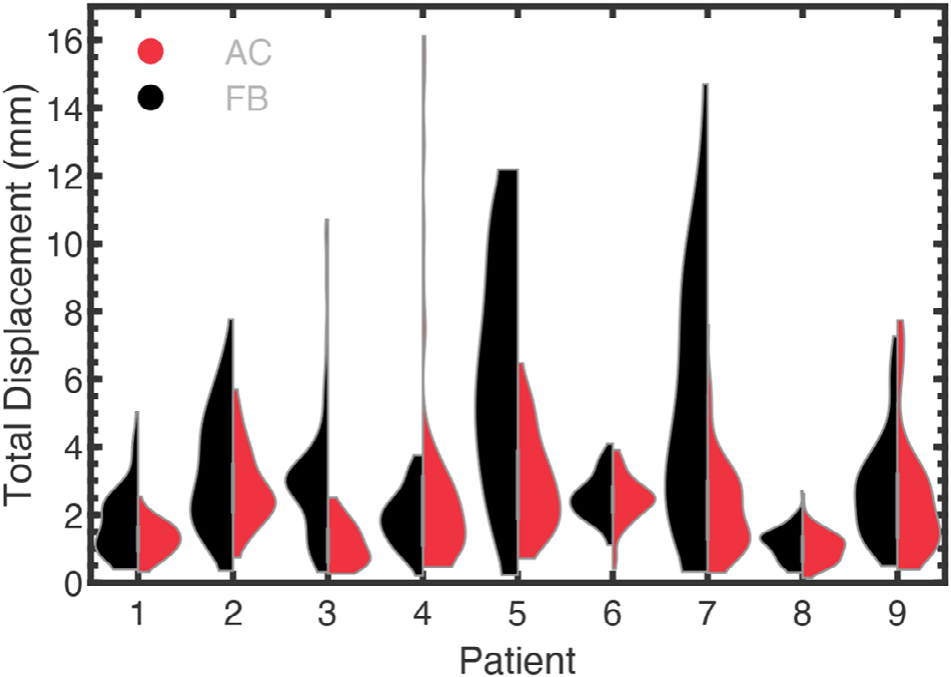
The magnitude of vector displacement of respiratory motion of the RV lead for each patient under (black, left‐half‐violin) free‐breathing (FB) and (red, right‐half‐violin) abdominal compression (AC) conditions.

The total displacement $\sqrt{RL^{2}+AP^{2}+IS^{2}}$ of the RV lead due to respiration under AC and FB is illustrated in Figure 5. The patient‐specific nature of respiration is demonstrated by each patient having unique distributions of respiration. Further, patients differ on the ability of AC to reduce respiratory motion. Generally, AC reduces the total displacement of respiratory motion, with 8/9 patients having a reduction in the mean RV lead total displacement. Notably, the patient that AC did not benefit, patient 4 (trace shown in Figure 1), had a single deep breath resulting in increased respiratory motion under AC. Averaged (± standard deviation) [95% CI] across patients, the mean 3D RV lead displacement was 3.2 ± 1.7 [1.9, 4.5] mm under FB and 2.2 ± 0.8 [1.6, 2.8] mm under AC.

**FIGURE 6:**
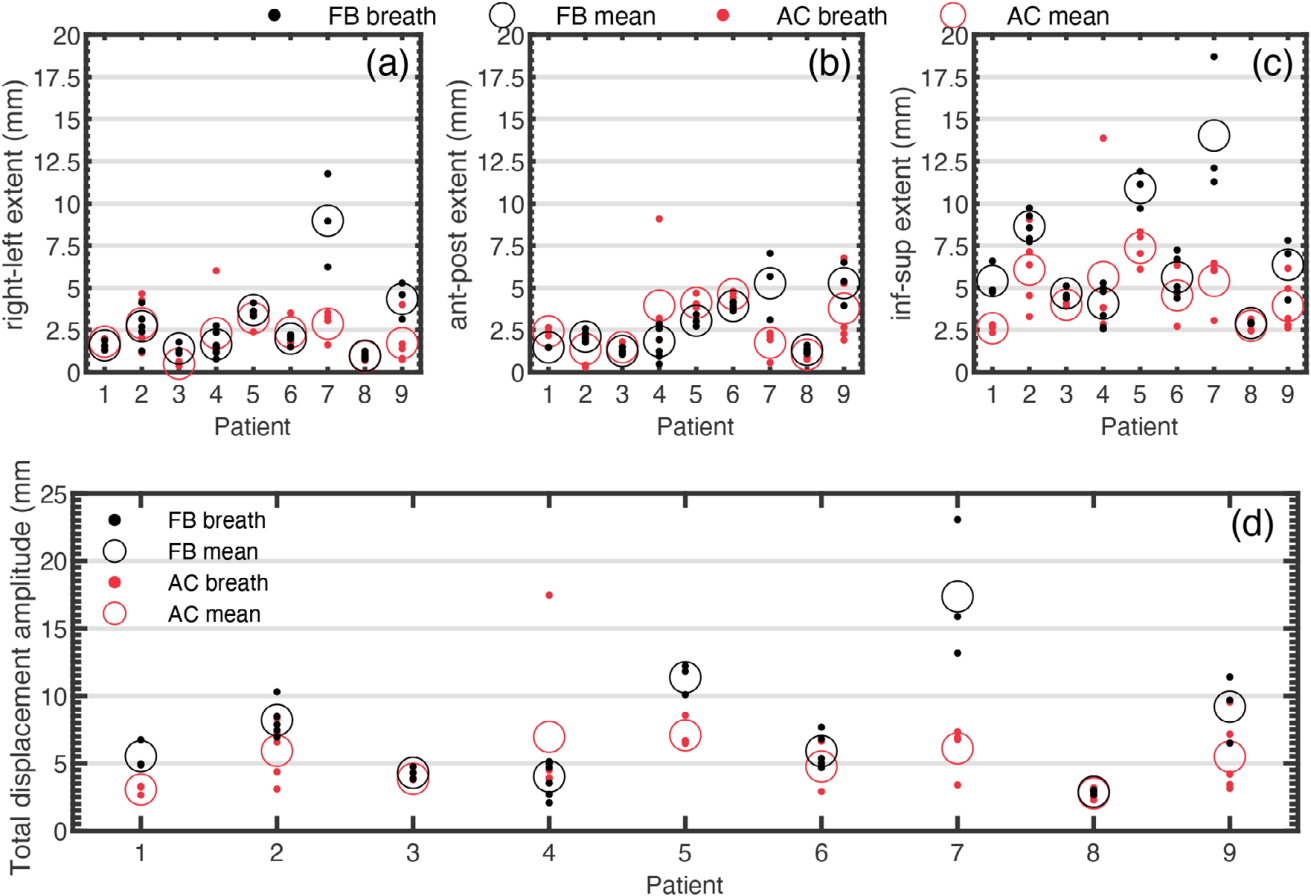
Peak‐to‐peak amplitude of respiratory motion for each patient's breaths (dots) under free breathing (black) and abdominal compression (red) in the (a) right‐left, (b) anterior‐posterior, and (c) inferior‐superior directions as well as for the (d) total 3D vector displacement. The mean amplitude across each patient's breaths is also shown as the circle markers.

Variability in respiratory‐induced RV lead motions between breaths is explored in Figure 6 by measuring the total displacement extent for each breath. Excluding patient 4, all patients had a reduction in the average extent of respiratory‐induced motion in the IS directions (Figure 6c) and for the total displacement motion trace (Figure 6d). In the RL (Figure 6a) and AP (Figure 6b) directions, the magnitude of respiratory‐induced motion is lesser, and the benefit of AC is less distinct. Averaged across patients, the mean (95% CI) extent of respiratory‐induced RV lead motion was [3.0 (1.1, 5.0) RL, 2.8 (1.6, 4.1) AP, 7.0 (4.2, 9.7) IS] mm under FB and [2.1 (1.4, 2.8) RL, 2.7 (1.7, 3.8) AP, 4.7 (3.5, 5.9) IS] mm under AC. Hence, the average (95% CI) respiratory‐induced RV lead motion extent was reduced by [1.0 (‐0.7, 2.6) RL, 0.1 (‐1.2, 1.4) AP, 2.3 (0.1, 4.5) IS] mm under AC compared to FB. The overall benefit of AC is accentuated by analyzing the amplitude of the total displacement motion trace, shown in Figure 6(d). Averaged across patients, the average (95% CI) total displacement amplitude was 7.6 (4.1,11.1) mm under FB and 5.1 (3.9, 6.3) mm under AC. Hence, the average (95% CI) reduction in amplitude of total respiratory displacement was 2.5 (‐0.5, 5.5) mm smaller with AC compared to FB.

Variations in respiratory motion for different positions in the heart are explored. Table 2 presents the average extent and average standard deviation of respiratory‐induced cardiac lead motion under AC and FB. Presented are the average extents across all nine patients with an RV lead as well as the subset of 6/9 patients with RV, LV, and RA leads. Compared to the RV lead, the LV and RA lead exhibit greater/lesser sup‐inf motion. Reduction in the magnitude of motion with AC compared to FB are consistent for the different leads, with variations in differences ≤1 mm.

**TABLE 2:** Average extent and standard deviations of respiratory‐induced motion in the [right/left, anterior/posterior, inferior/superior] directions under free breathing (FB) and abdominal compression (AC) conditions. Averages are reported for the RV lead of all patients and for the six patients with cardiac leads in the RV, LV, and RA.

	Average (± STD) extent (mm)		Average standard deviation (mm)	
	FB	AC	FB	AC
RV (*n* = 9)	[3.0 ± 2.5, 2.8 ± 1.6, 7.0 ±3.6]	[2.1 ± 0.9, 2.7 ±1.4, 4.7 ±1.6]	[1.3, 1.2, 3.0]	[0.9, 1.2, 2.0]
RV (*n* = 6)	[3.3 ± 3.0, 2.8 ± 1.9, 6.8 ± 4.1]	[1.9 ± 1.0, 2.2 ± 1.3, 4.6 ± 1.3]	[1.4, 1.2, 2.9]	[1.0, 1.1, 2.1]
LV (*n* = 6)	[2.1 ± 1.5, 3.0 ± 1.7, 8.4 ± 5.3]	[1.8 ± 0.8, 2.8 ± 1.1, 5.9 ± 2.6]	[0.9, 1.1, 3.5]	[0.8, 1.3, 2.8]
RA (*n* = 6)	[3.2 ± 1.5, 2.4 ± 1.4, 5.3 ± 2.8]	[2.1 ± 0.6, 1.8 ± 0.8, 3.8 ± 1.8]	[1.2, 1.0, 2.1]	[0.8, 0.8, 1.8]

## 4 DISCUSSION

As CR emerges as an experimental treatment for VT, research on accounting for motions from patient breathing and cardiac contraction have emerged. This work investigates the efficacy of a passive approach, AC, to reduce the magnitude of respiratory‐induced motion during treatment. By comparing respiratory‐induced motions of implanted cardiac leads in nine CR patients imaged with bi‐planar fluoroscopy under both AC and FB conditions, the efficacy of AC was investigated. Most (8/9) patients had reduced respiratory motions under AC, with the population average (95% CI) mean 3D displacement reducing by 1.0 (‐0.2, 2.2) mm under AC compared to FB. The patient who did not benefit from AC brought cognizance to the influence of intra‐fraction variability in respiratory motion, as a single deep breath skewed the metrics on the extent of patient breathing. Together, this work benchmarks the benefit of AC in a population of CR patients.

The simplest way to account for cardiorespiratory motion in CR is to expand the treated region using an internal target volume (ITV) margin to encapsulate the motions.^27^ This requires knowledge of the extent of respiratory‐induced motion. The average (95% CI) respiratory‐induced RV lead motion extent was reduced by [1.0 (‐0.7, 2.6) RL, 0.1 (‐1.2, 1.4) AP, 2.3 (0.1, 4.5) IS] mm under AC compared to FB. Consequently, ITV margins for respiratory‐induced motion would decrease by half of this peak‐to‐peak difference. There is challenge in defining ITV margins though, as individual patient breaths can vary in their amplitude, as illustrated in Figure 6. This highlights the importance of acquiring respiratory motion during simulation that is indicative of that during treatment. This effect was partially mitigated in this work by acquiring motion traces for multiple breaths and averaging them.

Another approach to account for cardiorespiratory motion in CR is the “mid‐position” approach, whereby margins to account for the dosimetric effect of errors, including motion induced errors, are used.^28^, ^29^, ^30^ In the van Herk et al. margin formula,^28^ the distribution of errors is used to calculate margins to offset their dosimetric effect. In the context of random intra‐fraction errors, the relevant metric is the standard deviation of the motions. These standard deviations are shown in Table 1, with most patients having smaller standard deviations of respiratory motion under AC. Thus, AC marginally reduces margins to account for respiratory motion in the IS direction, with minimal effect in the orthogonal directions. To our knowledge, no group has quantified the standard deviation of respiratory motion for CR patients.

This work adds to the emerging literature on the degree of respiratory motion in CR patients, warranting comparison with the existing literature. Prusator et al. analyzed respiratory‐binned CT images for 11 CR patients under AC^13^. In contrast to our work, where we filter out the higher frequency cardiac motion, respiratory‐binned CT may present artifacts such as blurring due to cardiac motion.^31^ This effect is smallest in the IS direction, where motion is predominantly from patient breathing (Figure 2), with our average IS amplitude under AC (4.7 ±1.6) mm in good agreement with the (4.7 ± 2.0) mm of Prusator et al.^13^ In the RL and AP directions, our reported respiratory motion amplitudes [2.1 ± 0.9 RL, 2.7 ±1.4 AP] mm overlapped with the [3.9 ± 1.6 RL, 4.1 ± 0.8 AP] reported by Prusator et al. Our results being smaller than Prusator et al. may be explained by cardiac motions which predominantly occur in these directions. Our results under FB can be compared to the cardiorespiratory motion of the ICD lead tip for 20 patients as reported by Knybel et al.^21^ They found amplitudes of cardiorespiratory motion to be [3.4 ± 1.9 RL, 3.1 ±1.6 AP, 5.0 ±2.6 IS] in comparison to [3.0 ± 2.5 RL, 2.8 ±1.6 AP, 7.0 ±3.6 IS] found in our work. Finally, Roujol et al. found the average amplitude of respiratory motion to be [3.3 ± 1.2 RL, 3.6 ± 1.2 AP, 7.0 ± 1.8 IS] for 27 patients under FB using electro‐anatomical mapping.^16^ This work uses the same method of respiratory motion extraction as our work and provides excellent agreement with our results under FB of [3.0 ± 2.5 RL, 2.8 ±1.6 AP, 7.0 ±3.6 IS]. Together, our results show consistency with the existing literature and add to the literature on the extent of respiratory motion in CR patients.

Previous work has investigated the impact of AC on the heart's respiratory motion. Mannerberg et al. analyzed the center of mass shift from max expiration to max inspiration for different heart structures using four‐dimensional CT (4DCT) images for 18 lung cancer patients imaged under both AC and FB.^22^ They found AC decreased the median respiratory motion of heart structures by 1–3 mm. This is consistent with our work, with an average (± STD) reduction in amplitude of total respiratory displacement of the RV lead of (2.5 ± 3.9) mm, with a median reduction of 2.3 mm. Cecchi et al. analyzed 4DCT for 18 lung cancer patients under FB and 18 liver cancer patients under AC.^23^ In addition, three VT patients had 4DCT's acquired under both FB and AC. The difference in median magnitude of COM translation (FB‐AC) ranged from ‐0.8 mm to 2.1 mm for different heart structures but with no statistically significant differences (*p* ≤0.05) found. For the three VT patients, changes in the COM translations under FB and AC were patient, direction, and structure specific. Our work extends the understanding of AC's effect on respiratory motion for CR patients by acquiring imaging under both AC and FB conditions, with the respiratory trace of multiple (3–5) breaths extracted. Variability in the amplitude of respiration across individual breaths is noted and evaluated, which cannot easily be extracted from a 4DCT image. Together, our results add to the literature to suggest that AC moderately reduces the magnitude of respiratory motion for most CR patients.

While AC presents a relatively simple, easy to implement, solution to manage respiratory motion for CR, it is only a small portion of the overall options for motion management in CR. In addition to respiratory motion, cardiac motions must be quantified and accounted for to ensure the target receives the intended dose through its overall motion. One solution is to account for both cardiac and respiratory motions using margins.^27^, ^29^, ^30^ Alternatively, investigations into active motion management in CR have been published. Cardiac gating has been proposed and demonstrated,^32^, ^33^, ^34^ with its impact on treatment volumes explored.^35^ Multi‐leaf collimator tracking has also been presented, in the context of CR for atrial fibrillation.^14^ Work has also demonstrated real‐time motion management on an MR‐linac.^36^, ^37^ Another option is to deliver treatment under breath‐hold.^38^ While many promising methods of motion management in CR have been presented, it remains to establish the clinical significance of each method.^39^

Bi‐planar kV fluoroscopy was used to track the 3D position of cardiac leads for 15–20 s at 5 Hz, enabling extraction of the respiratory component of the motion. The largest source of uncertainty in motion measurement is tracking the cardiac lead tips in each bi‐planar fluoroscopy image. This uncertainty is reduced when the tip is triangulated, due to the combination of two measurements, and when lowpass filtering to extract the respiratory component of motion, which reduces high frequency tracking errors. We estimate average uncertainties in motion tracking to be <0.5 mm based on phantom simulations. Bi‐planar fluoroscopy was incapable of tracking the radiolucent target, which is a limitation of this work. Instead, cardiac leads were used as a surrogate for the target's respiratory motion. The suitability of cardiac leads as surrogates for respiratory motion has previously been investigated.^13^, ^26^ While bi‐planar fluoroscopy is limited in resolving the targets motion, its use is also a strength of this work. First, it enables tracking cardiac leads at 5 Hz, which permits extracting respiratory motions from cardiorespiratory motion with multi‐band filtering.^16^ Second, it allows for imaging of multiple breaths, permitting a deeper understanding of the characteristics of patient breathing. However, acquiring data over an extended duration is warranted to assess the overall intra‐fraction variability in breathing during treatment. In contrast, respiratory 4DCT can present artifacts from irregular breathing and cardiac motion.^31^ MR imaging has the potential to resolve respiratory motions but is challenged by metal artefacts from the cardiac leads that CR patients have.^25^

Analyzing the respiratory‐induced motion for the tip of each patient's cardiac lead permits comparison of respiratory motions for different cardiac regions. Table 2 illustrates differences in the extent and standard deviation of respiratory‐induced motion for the six patients with three implanted cardiac leads. Typically, the LV and RV leads are in closest proximity to CR targets. Here, the LV lead had a moderately larger average extent of IS respiratory‐induced motion of about 1–3 mm compared to the RV and RA leads. Generally, the respiratory‐induced motion of the heart can be approximated as a translating and rotating rigid body,^40^, ^41^ which may explain these differences.

Further work is needed to establish the role of AC in CR. First, analysis on a larger patient cohort is warranted. Currently, this is challenging as CR is a relatively new modality, with limited patients being referred for treatment. Second, imaging data could be acquired over longer durations, including exploring the effect of inter‐fraction variabilities in breathing between acquiring imaging for treatment planning and the day of treatment (CR is typically delivered in a single fraction). Ideally, extended imaging durations enable for a full understanding of the intra‐fraction motions expected throughout treatment. In practice, this is challenged by the high temporal resolution required to separate cardiac and respiratory‐induced motions, and the need to keep imaging dose as low as reasonably achievable. Such data would yield insight on the representativeness of a subset of motion data to that seen throughout treatment. For example, when the deep breath of patient 4 under AC is removed from the analysis, all patients benefit from AC (quantified by the mean 3D displacement), but removing this breath from the analysis is non‐trivial without knowing its representativeness of that during treatment. Another important consideration to explore is if AC pushes nearby organs closer to the target. For example, the stomach can be a dose‐limiting organ, and it has been observed that AC brings the stomach closer to the heart for a patient,^42^ although the opposite effect has also been reported.^23^

## 5 CONCLUSIONS

AC reduces the respiratory‐induced 3D motion of the cardiac leads in the majority of the investigated radioablation patients. Further work on an expanded patient population, with volumetric data, and prolonged imaging times is warranted.

## AUTHOR CONTRIBUTIONS

All authors contributed to the conception and design of the study and contributed to data acquisition. JM analyzed and interpreted all data and wrote the manuscript. All authors critically reviewed and revised the manuscript.

## CONFLICT OF INTEREST STATEMENT

Steven Thomas, Devin Schellenberg, and Marc W. Deyell report research funding from Varian Medical. Steven Thomas reports a patent on motion synchronized arc therapy.
